# The Interaction between Interoceptive and Action States within a Framework of Predictive Coding

**DOI:** 10.3389/fpsyg.2018.00180

**Published:** 2018-02-20

**Authors:** Amanda C. Marshall, Antje Gentsch, Simone Schütz-Bosbach

**Affiliations:** General and Experimental Psychology Unit, Department of Psychology, Ludwig Maximilian University of Munich, Munich, Germany

**Keywords:** predictive coding, motor control, embodied selfhood, interoception, agency

## Abstract

The notion of predictive coding assumes that perception is an iterative process between prior knowledge and sensory feedback. To date, this perspective has been primarily applied to exteroceptive perception as well as action and its associated phenomenological experiences such as agency. More recently, this predictive, inferential framework has been theoretically extended to interoception. This idea postulates that subjective feeling states are generated by top–down inferences made about internal and external causes of interoceptive afferents. While the processing of motor signals for action control and the emergence of selfhood have been studied extensively, the contributions of interoceptive input and especially the potential interaction of motor and interoceptive signals remain largely unaddressed. Here, we argue for a specific functional relation between motor and interoceptive awareness. Specifically, we implicate interoceptive predictions in the generation of subjective motor-related feeling states. Furthermore, we propose a distinction between reflexive and pre-reflexive modes of agentic action control and suggest that interoceptive input may affect each differently. Finally, we advocate the necessity of continuous interoceptive input for conscious forms of agentic action control. We conclude by discussing further research contributions that would allow for a fuller understanding of the interaction between agency and interoceptive awareness.

## Introduction

Recent years have seen a resurgence of scientific interest in the foundations of selfhood, our ability to perceive and maintain a conscious sense of self (Hohwy, 2007; Zahavi, 2008; Blanke and Metzinger, 2009). The common consensus postulates that selfhood is grounded in bodily processes, suggesting that it arises from a conjoined processing of bodily signals and afferent perceptual input (Bermúdez, 1995; Gallagher, 2005). However, the vast majority of research has focused on the way motor signals contribute to the emergence of selfhood (Roessler, 2005; Pacherie, 2008; Gallese and Sinigaglia, 2010), while disregarding the role of interoceptive input. Interoception refers to the awareness of internal bodily states such as hunger, heartrate, a general sense of arousal or pain or muscular and visceral sensations which are achieved by processing homeostatic signals transmitted by the autonomic nervous system (Critchley et al., 2004; Garfinkel et al., 2015). Interoception is thus distinct from exteroception (the intake and processing of environmental information via sensory organs) and proprioception (a sense of the body’s position within an external environment). An important characteristic of interoceptive signals is that they are transmitted continuously. In combination with motor signals, they may thus form a core building block of selfhood which is likewise experienced as a seamless phenomenological state. Here, we aim to provide an insight into the interaction between interoceptive awareness and action perception as the two potential cornerstones leading to the experience of bodily selfhood.

We will begin by introducing the concept of predictive coding as the mechanistic process thought to underpin both interoception and motor experience, thus forming an initial, theoretical link between both concepts. We will proceed to discuss empirical work suggesting a functional interplay of interoceptive and motor signals in the realms of sensory attenuation and action control, as well as elaborating on the neuroanatomical architecture suggesting reciprocal connections between structures linked to interoceptive and motor processing. We will then offer three proposals detailing the specific functional relationship between interoception and motor actions. Our suggestions for this interactive mechanism will highlight the role of interoceptive predictions for the generation of motor states and address pre-reflexive (implicit) and reflexive (explicit) modes of action control. In addition, they will differentiate between the roles of transitory motor signals and continuous interoceptive input. Finally, we will touch on the potential impact of social interactions for generating the mental frameworks used to form an understanding of self and self-related motor actions. We will conclude by discussing the implications of considering a joint contribution of interoception and action to the generation of conscious selfhood.

## Embodied Selfhood: Predictive Coding for Interoception and Motor Experience

Recent contributions from cognitive science and neuroscience have led to a major theoretical advance in the field of embodied cognition by implicating the contribution of ‘predictive processing’ in the generation of selfhood and its accompanying constituents such as a feeling of agency and an awareness of one’s own internal states (Friston, 2002). To date, several accounts postulate that top–down predictions about the sensory consequences of events shape their perception, the generation of selfhood and the general cognitive framework for perceiving and acting within the environment (Metzinger, 2003, 2004, 2005; Allen and Friston, 2016). In general, neuronal representations in higher hierarchical levels are thought to generate predictions of representations at lower levels. These predictions are subsequently matched to lower-level representations constructed from sensory input, thereby generating a prediction error signal. This mismatch signal travels back up the hierarchy where it is used to update higher-order representations. This exchange of signals is thought to occur on multiple levels, thereby generating a hierarchically structured explanation of sensory input.

The idea of predictive hierarchical models has long been considered a fundamental mechanistic underpinning of motor experience. To account for the phenomenology of agency, initial forward models based on corollary discharge (Blakemore et al., 2002; Gallagher, 2005; Frith, 2012) have been extended to consider the experience of agency as a consequence of outcome predictions formed by hierarchical generative models (de Vignemont and Fourneret, 2004). For example, Hohwy (2007) postulates that predictive models for agency may constitute one instance of the brain’s overall cognitive system for representing and attending to cognitive and motor states (for reviews see Friston, 2002; Kersten et al., 2004). Accurately predicted sensory feedback is thought to be attenuated, thereby producing a feeling of ‘mineness’ concerning the action and circumventing the ambiguity of relying purely on intentions, as only feedback from predicted, self-initiated movement is attenuated. Accounts in line with this idea have proposed that disturbances of agency, as experienced in Schizophrenia, are caused by imprecise predictions about the sensory consequences of actions (Frith, 1987; Blakemore et al., 2000a; Frith et al., 2000; Synofzik et al., 2010; Voss et al., 2010). Of particular note is that a generative explanation of agency is a necessary assumption for active inference which in itself forms a central component of popular predictive coding accounts such as the free energy principle (Friston and Stephan, 2007; Hohwy, 2007, 2010; Friston, 2009; Brown et al., 2013). A prerequisite for modulating one’s actions to bring about sensory input in accordance with model predictions (i.e., minimize free energy) is the ability to predict which actions will lead to a better model confirmation.

Predictive coding has likewise been attributed to interoception. As early as 1981, Pennebaker and Skelton demonstrated that perception of bodily sensations was strongly modulated by participants’ prior beliefs (Pennebaker and Skelton, 1981). However, despite this early observation, interoception was long considered a purely bottom–up, sensory driven phenomenon. Recently this view has changed. The idea of top–down processes for the generation of interoceptive states was borrowed largely from the exteroceptive domain. Here, the theory of hierarchical predictive models that follow the rules of Bayesian inference was originally proposed to account for the perception of stimuli originating outside of the body (Friston, 2005; Friston et al., 2009; Adams et al., 2013). Predictive generative models applied to interoception likewise postulate the minimization of prediction error as a mechanism for self-representation (Seth et al., 2012; Suzuki et al., 2013). Specifically, self-consciousness is thought to be based on interoceptive feeling states emerging from the interactions of predictions and prediction error (Critchley and Seth, 2012). For example, work exploring a subjective sense of self within a virtual environment (Nahab et al., 2011) has suggested that it is the result of successful matches between expected and received sensorimotor signals.

A concrete account of the way in which predictive coding may contribute to interoception was recently offered by the interoceptive predictive coding model. Developed by Seth et al. (2012) and extended over subsequent years (Seth, 2013; Seth and Friston, 2016), it provides the first theoretical framework of this account which can be subjected to empirical investigation. Importantly for the topic of this paper, it also tentatively highlights the interplay between interoception and action experience. Seth and colleagues’ model includes an “agency component” and a “presence component” which are subdivided into a state and an error module. Within this framework, the presence component reflects processes related to interoceptive information whereas the agency component reflects motor control processes, including forward model and error monitoring mechanisms that underlie the sense of agency. The model postulates that control signals are generated within the state modules of both components. These signals are passed on to the autonomic system by the presence component and the sensorimotor system by the agency component. They are accompanied by prediction signals transmitted to the error module which presage the outcome of this signal (Sommer and Wurtz, 2008). In the error module, the prediction signal is compared to afferent feedback transmitted by the sensorimotor (agency component) or autonomic system (presence component). The extent of match or mismatch between the predicted and afferent signal determines the size of an error signal which is sent back to the state module (Paulus and Stein, 2006). According to the model, a sense of agency or presence is achieved when informative predictions are successfully matched to afferent inputs, thereby suppressing the error signal. Conversely, a large error signal due to imprecise or misinformed predictions leads to a reduced sense of presence or agency. The model emphasizes the interaction between motor experience and interoception by postulating that the overall heightened or lowered interoceptive state will affect the signals passed from the agency state to the sensorimotor system. In addition, the agentic predictions about sensorimotor outcomes are thought to contribute to the overall interoceptive state.

Within the motor domain, the nature of predictive elements governing action perception has been well defined. Motor signals are thought to be accompanied by a so-called efference copy (Wolpert and Flanagan, 2001). This copy of the motor command is processed in relevant sensory hubs of the brain to anticipate the sensory consequences of an action whose perceived intensity is thereby reduced (Shergill et al., 2003). Applying this concept to interoception suggest that the same form of predictive modeling occurs for interoceptive, autonomic signals. Evidence to this effect comes from a recent study by Salomon et al. (2016) who reported that presenting a visual stimulus in tune with participants’ heartbeat resulted in a reduced neural measure of its exteroceptive perception and a decreased likelihood of its conscious detection. The authors’ findings thus offer a tentative indication for a similar predictive modeling of the interoceptive heartbeat signal which produces the well documented suppression effect for exteroceptive stimuli that are presented in an anticipated pattern or manner. A predictive modulation of the heartbeat signal also fits within the confines of simulation theory which has been used to suggest that the brain makes predictive forecasts of affective reactions to future events to guide actions in the present (Gilbert and Wilson, 2009). With respect to the heartbeat, reports in this domain have highlighted that mental simulation of movement affects heart rate and pulmonary ventilation as a function of imagined effort (Decety et al., 1991), thereby demonstrating that mental extrapolations have a direct impact on interoceptive signals.

## The Interaction Between Action Representation and Interoception

Past work has highlighted a potential link between interoception and motor actions, discussing the successful processing of interoceptive signals as a prerequisite for hierarchically structured forms of motor control to achieve anticipated interoceptive states (Edwards et al., 2012; Pezzulo et al., 2015; Limanowski, 2017). However, this work has predominantly focussed on action implementation (e.g., active inference) while neglecting the way interoceptive processing may contribute to the perception and subjective experience of actions (e.g., agency). The following section will elaborate on the link between agency and interoception by discussing several lines of evidence detailing an effect of motor-actions on the construction of interoceptive awareness and a reverse influence of interoception on action perception.

### Co-occurring Impairments of Motor and Interoceptive States

Past work has shown that disorders of motor and interoceptive states often (but not exclusively) occur simultaneously (Robertson, 2000; Sumner and Husain, 2008; Ruhrmann et al., 2010; Sierra and David, 2011). Clinical research to this effect has demonstrated that disorders such as Tourette syndrome, schizophrenia and depersonalization disorder often disrupt both feelings of selfhood and action authorship (Seth, 2013; Sedeño et al., 2014). For example, Ardizzi et al. (2016) reported a relation between altered sensorimotor processes and interoceptive accuracy in a cohort of patients suffering from schizophrenia. Based on the observation that schizophrenic patients had lower interoceptive accuracy, as well as a distorted sense of body and action ownership, the authors suggested that both agency and interoceptive states suffered from a loss of basic, pre-reflexive aspects of selfhood. However, while disorders of agency and interoceptive states often occur in conjunction, this is not always the case. As such, not all schizophrenic patients report a disturbance of conscious selfhood (Ruhrmann et al., 2010) and studies of disorders such as the Alien Hand syndrome demonstrate that depersonalization and derealisation do not necessarily have to impact on agency (Sumner and Husain, 2008). Hence, a closer look at underlying sub-mechanisms and associated phenomenological markers supporting the interaction between agency and interoception is warranted.

A further population of interest in this regard are individuals suffering from deafferentation, the selective loss of cutaneous touch and proprioception. Deafferentation has been shown to affect motor experience (Bosbach et al., 2005; Balslev et al., 2007). Crucially, deafferentation should have a significant impact on predictive mechanisms thought to underlie the generation of agency, as the absence of afferent sensory input no longer allows the comparison of predictions with proprioceptive feedback. In support of this, past work has highlighted that patients with deafferentation are significantly impaired at generating a sense of agency and adjusting to action outcomes. For example, Hermsdörfer et al. (2008) reported that an individual with chronic deafferentation of the trunk and limbs (I.W.) employed excessive grip force of a hand-held object and was unable to adjust this according to anticipated load magnitude. I.W. was also unable to differentiate his own from computer generated actions when relying on proprioceptive information alone (Balslev et al., 2007). At a more complex cognitive level, he was unable to interpret another person’s expectation of weight when seeing him lift boxes (Bosbach et al., 2005). In extreme cases (for example high spinal cord injury) deafferentation has also been shown to affect interoception when both afferent and efferent pathways are impaired. In this regard, Pistoia et al. (2015) found that individuals with sensory deafferentiation resulting from high spinal cord injury were unable to judge their own emotional response to scenes eliciting fear and anger despite being able to recognize the emotions in others. Findings in this regard highlight the importance of sensory input for generating internal states as well as for adjusting motor actions to perform efficient movements. They further highlight the importance of considering the contributions of afferent and efferent pathways to generate these states, however, a detailed description of this lies outside the scope of this paper.

### Motor States Influence Interoceptive and Sensory Processing

A more causal line of evidence demonstrates that experimental manipulation of perceived agency can influence reported presence in both normal individuals and schizophrenic patients (Lallart et al., 2009). To this effect, Gutiérrez-Martínez et al. (2011) demonstrated that participants had higher pain tolerance if they were able to manipulate an avatar in virtual reality who was immersed in a pleasant and peaceful environment. Based on their findings, the authors argued that agentic control within this peaceful, non-painful reality enabled participants to increase their sense of presence (here defined according to the Virtual Reality literature as true immersion in the virtual environment) to thereby reduce the simultaneous painful experience in real life.

Input from motor-related states to interoception is also demonstrated by past work highlighting sensory attenuation of self-generated actions, in particular the proximal tactile consequences of actions. For example, participants consistently report reduced sensory perception of self-generated tickle or applied force (Weiskrantz et al., 1971; Claxton, 1975; Shergill et al., 2003). In a series of manuscripts, Blakemore and colleagues have attributed this phenomenon to internal forward models that enable a comparison of predicted sensory consequences of movements to afferent feedback (Blakemore et al., 1998, 1999, 2000b). Interestingly, observations from these imaging studies suggest that rather than being exclusively exteroceptive phenomena, sensory attenuation effects of self-produced tickle sensations may also result from interoceptive feedback as a consequence of simultaneously activated affective brain systems. Results show that cortical activity increases not only in the secondary somatosensory cortex but also in the anterior cingulate gyrus when subjects experience externally produced relative to self-produced tactile stimulation (Blakemore et al., 2000b). The anterior cingulate cortex has been implicated in affective behavior (e.g., Vogt et al., 1992) and is consistently activated together with interoceptive brain areas during performance monitoring (e.g., Klein et al., 2007; cf. discussion below). Hence, there is reason to assume that the reduced motor experience error signal for a self-generated and thus accurately predicted action is also processed by the interoceptive error unit, where it may contribute to generating attenuated interoceptive (i.e., tickle) sensations. In particular, the human tactile system, consisting of fast-conducting afferents (Aβ) and slowly conducting unmyelinated afferents (C), serves a dual function by providing both sensory-discriminatory information and affective-motivational qualities. The latter involves feelings from the body, including pain, temperature, itch, sensual touch that have been shown to be processed by neural pathways strongly implicated for interoceptive processing (cf., Craig, 2002).

Further support for the effect of motor predictions on interoceptive sensations generated by tactile stimulation comes from studies on affective touch involving so-called C-tactile (CT) afferents. Unmyelinated CT afferents in hairy skin have been found to be specifically tuned to human caress and to directly project to the insular cortex (Olausson et al., 2002), where the pleasantness of these sensations correlates with the degree of insular activation (Löken et al., 2009). Importantly, action has been found to reduce the intensity of these sensations when they are self-generated as compared to externally applied (Guest et al., 2009). Moreover, a recent study manipulating agency for CT optimal stimulation during interpersonal touch (Gentsch et al., 2015) showed that predictions about interoceptive states are generated during self-generated actions and can change haptic softness perception of another individual’s skin. These results suggest that agency involves the simulation of interoceptive sensations (i.e., not only proprioceptive and exteroceptive sensations), which can in turn amplify the haptic sensory pleasure derived from the active touch of others. Blakemore and colleagues hypothesize that this prediction-based modulation of sensory information processing might facilitate the identification of self-generated actions and underlie the distinction between one’s own self and others, thereby contributing to a sense of selfhood (Blakemore and Frith, 2003). This idea corresponds to past work exploring the link between impaired motor predictions and resulting feelings of incompleteness in Obsessive Compulsive Disorder (OCD). To this effect, Gentsch et al. (2012) reported electrophysiological evidence indicating that OCD patients suffer from an inability to suppress the sensory consequences of their own actions which they likewise attributed to impaired forward model mechanisms. Most importantly, the authors suggested that the increased mismatch between predicted and actual outcomes coincided with feelings of incompleteness (i.e., distressing sensations of things not being quite right and completed). Crucially, extensions of the original interoceptive predictive coding model introduced above have emphasized the inclusion of emotional and affective states in interoceptive processing. Working from the premise that feelings of incompleteness, as an affective state, are likewise an interoceptive phenomenon, the results of Gentsch and colleagues once more indicate that an impaired predictive mechanism relating to agency may influence the extent of awareness of internal signals (see **Box 1** for a more in depth discussion of interoceptive predictive coding and affective states).

The relation between emotions, interoceptive states and motor actions. The assumption that interoception exerts a direct influence on action representation raises the question how different emotional states and awareness of these influence agentic input to action selection. Intriguingly, recent evidence from Marshall et al. (2017) reports that events of different valence modulate markers of interoceptive states in distinctly different ways. While neutral stimulus repetitions were found to elicit elevation of the Heartrate Evoked Potential (HEP) component, repetitions of negative stimuli were found to produce a significant decrease of HEP amplitude. Increased amplitude of the Heartrate Evoked Potential has been widely interpreted as an indication of increased embodied self-awareness (Critchley et al., 2004; Pollatos and Schandry, 2004; Pollatos et al., 2005). The authors’ findings thus suggest that while neutral events may lead to an increase of interoceptive states, negative events reduce sensitivity to internal states. Several papers have argued that interoceptive awareness is a prerequisite for conscious forms of action regulation and error monitoring (Ullsperger et al., 2010; Prinz, 2012; Sueyoshi et al., 2014). Following this argument, lowered interoceptive awareness, signaled by a lower HEP after experiencing a negative event, would lead to reduced conscious control of actions. However, this interpretation runs contrary to findings in the cognitive literature where experiences of negative events and depressed mood are associated with increased executive control and more elaborate processing of information (Mackie and Worth, 1991; Schwarz et al., 1991; Bodenhausen et al., 1994). The cognitive literature interprets this phenomenon as an increased focus on external events to speedily adapt to a hostile environment and prevent more negative outcomes. This interpretation is also offered by Marshall and colleagues who propose that negative events may lead to a reduced internal focus (lower HEP) thus freeing up attentional resources to an adverse and challenging environment. Interpreting reduced HEP amplitude as lowered interoception is therefore problematic if working from the premise that it is required for conscious forms of action regulation (Wessel et al., 2011; Godefroid et al., 2016). Similarly, Seth et al.’ 2012 interoceptive predictive coding model postulates that ‘a sense of presence arises when informative interoceptive prediction signals are successfully matched to inputs so that prediction errors are suppressed’ (p. 3). This suggests that reduced interoception (i.e., reduced awareness of bodily signals) is the result of large error signals resulting from imprecise or inaccurate predictions which do not match the true sensory feedback provided by the autonomic nervous system. However, this is not the case for the results of Marshall and colleagues’ experimental scenario in which reduced HEP amplitude is the product of a match between actual and (accurately) predicted outcomes for repeated negative events. Thus, reduced HEP amplitude in this scenario may not indicate reduced awareness of bodily signals. Instead it may signal the dissociation of the internal self from negative outcomes which possibly promotes increased external focus to remedy a negative state and attend to a hostile environment. Marshall and colleagues’ findings thus suggest that the construction of interoceptive awareness may be more complex than originally assumed. Rather than arising purely from a match or mismatch between predicted and afferent sensory signals their observations highlight that decreased or increased interoceptive awareness may depend on multiple factors such as the type of interoceptive state that is being projected and compared. This highlights the importance of carefully considering the experimental scenario when interpreting the meaning of markers for internal states, as well as the need to explore the way events of different valence may influence interoceptive sensitivity and its effects on motor actions.

### Interoception Contributes to Error Awareness and Adaptive Behavior

A primary line of evidence which suggests input from interoception to action-related states is provided by work on performance monitoring. For example, Ullsperger et al. (2010) argue that interoceptive awareness is a prerequisite for conscious error perception and the resulting implementation of behavioral adjustments. The authors center this argument on the anterior insula cortex (AIC), which forms part of the ‘salience network’ in conjunction with the medial frontal cortex (pMFC) and the frontal operculum. This system is linked to processing motivationally important information in affective and cognitive domains (Peyron et al., 2000; Bartels and Zeki, 2004; Ramautar et al., 2006; Lamm and Singer, 2010). In addition, the AIC is commonly viewed as a monitor and regulator of the body’s homeostatic signals and interoceptive states (Craig, 2002, 2009) via its functional connections to the autonomic nervous system (Matthews et al., 2004). Based on this, the authors suggest the AIC may serve as a platform to conscious error awareness by acting either as a monitor or an active agent eliciting an error orienting response in the autonomic nervous system. The perception of this error related arousal pattern is thought to produce conscious error awareness which in turn enables the organism to recruit mental and physical resources to perform behavioral adjustments. The authors develop their argument across several lines of research: in conjunction with other known systems, the anterior insula is consistently activated during performance monitoring. In a meta-analysis of 55 fMRI studies, Klein et al. (2007) report pMFC and AIC activation across multiple task conditions calling for behavioral adjustments. These included pre-response conflict, decision uncertainty, response errors and negative feedback. Crucial for the authors’ claim that the AIC forms part of a chain leading to the recruitment of adaptive resources was the selective activation of the AIC during tasks calling for different kinds of behavioral adaptation. Thus, while conditions involving the prevention of errors (i.e., risk prediction) activated the superior AIC, conditions involving the adaptation to a committed error (i.e., negative feedback) correlated with inferior AIC activation. This highlights the possibility that sub-regions of the AIC respond selectively to behavioral adjustments demanded by different tasks. Contrary to other structures associated with error processing, studies have also shown that the anterior insula is selectively engaged for conscious but not subconscious errors (Hester et al., 2005; Klein et al., 2007). It has also been discussed as an indirect contributor to other known correlates of conscious error perception such as the electrophysiological Pe component (Klein et al., 2007). Indications that perceived increases of autonomic arousal underpin the conscious perception of errors come from studies contrasting autonomic activation to conscious and subconscious errors. They report increases in skin conductance (O’Connell et al., 2007), heart rate deceleration and pupil diameter (Wessel et al., 2011) toward erroneous relative to correct responses for consciously but not subconsciously committed errors. In addition, the AIC has been found to increase functional connectivity to other saliency network structures in the somatosensory cortex during error awareness which has been interpreted as an effort to amplify the salience signal of a detected error (Ullsperger et al., 2010). Evidence for the link between increased arousal signals and behavioral adjustment comes from studies highlighting that activation of the salience network coincides with improved task performance (Weissman et al., 2006; Boly et al., 2007) as well as from clinical observations which have attributed pathological alterations of error awareness in autism, attention deficit hyperactivity disorder and addiction to altered activity in the AIC (Paulus and Stein, 2006; Hester et al., 2007; Silani et al., 2008; Vlamings et al., 2008; O’Connell et al., 2009; Paulus and Stein, 2010). Ullsperger and colleagues’ line of reasoning rests on correlative rather than causative evidence and will benefit from subsequent research studying error awareness and arousal patterns among patient populations with focal lesions to the anterior insula to test the merit of this claim. However, the AIC’s monitoring function of interoceptive, homeostatic states meets a common consensus. Suggesting this as a platform toward conscious error perception and subsequent behavioral adaptation thus forms a plausible suggestion and provides an indication for the functional importance of an interplay between interoception and motor experience.

Electrophysiological evidence for the contribution of interoception to error detection comes from work undertaken by Godefroid et al. (2016) who conducted a Go/No-Go task in which they explored the relation of interoceptive awareness to electrophysiological correlates of conscious error perception. The authors reported that the error positivity (Pe) component’s amplitude to aware errors correlated positively with interoceptive awareness measured through a heartbeat detection task. Interestingly, this correlation emerged only for trials in which participants could see their responding hand and not for trials in which the hand was obscured. Godefroid and colleagues’ findings thus provide further evidence for interoceptive input to motor adaptation and further indicate that interoceptive awareness may have to interact with further sources of information to enable error perception to reach conscious perception. These findings correspond to work presented by Sueyoshi et al. (2014) who conducted a similar investigation in which they related interoception to error monitoring during a Simon task while recording the amplitudes of the error related negativity (ERN) and error-positivity (Pe) components. Results showed that participants’ scores on the heart rate detection task correlated with Pe amplitude irrespective of stimulus valence while the correlation between the ERN and heart rate score occurred only for emotionally significant stimuli (disgust stimuli). Of particular interest for the proposition that interoception influences action-related states was that heart rate detection scores were also correlated with the degree to which reaction times slowed after error commission, thereby forming a further indication that interoception contributes to behavioral adaptation and regulation.

### Interoception for Effective Self/Action-Regulation

The link between interoception and action regulation suggested by the work of Sueyoshi et al. (2014) is echoed in reports linking greater interoceptive sensitivity (heightened perception of interoceptive signals measured by a heartbeat tracking task) to better self-regulation and pain tolerance. The subjective experience of pain has been shown to depend heavily on top-down influences such as expectations and attention (Fardo et al., 2017). Thus, regulatory capacity has the potential to greatly reduce its magnitude. Investigating a sample of healthy individuals and somatoform patients, Weiss et al. (2014) found that greater interoceptive sensitivity corresponded to higher amounts of self-reported regulation capacity. In addition, somatoform patients showed decreased interoceptive sensitivity which coincided with a lower threshold for pain. Similarly, Herbert et al. (2007) reported that good heartbeat perceivers with high interoceptive sensitivity showed more effective self-regulation of physical load during an exercise task than poor heartbeat perceivers.

However, despite empirical accounts linking interoception to action regulation, heightened interoception is not always related to greater action control. Interestingly, for intuitive or subconscious processes, higher levels of interoception can lead to more impulsive actions. For example, Ainley et al. (2014) reported that interoceptive awareness is linked to a higher likelihood of automatic imitation. The authors found that good heartbeat perceivers had greater difficulty in inhibiting the tendency to imitate an observed action and suggested that high interoceptive awareness may produce a stronger internal representation of action consequences which leads to higher motor reactivity to observed actions. Similarly, Dunn et al. (2010) discovered that increased interoceptive awareness could either facilitate or hinder intuitive decision making depending on whether perceived bodily signals suggested advantageous or disadvantageous choices. Findings to this effect highlight that interoceptive input to motor representations may result in increased action regulation for conscious processes conducted over longer time frames while leading to more impulsive actions for transitory, subconscious processes.

## A Perspective on Integrating Action Representation and Interoception

The evidence discussed thus far speaks to the link between action prediction and the active generation of interoceptive states. It also highlights the reverse relationship by detailing a contribution of interoception to motor experiences which has remained unaddressed in the literature to date. In the following section, we elaborate on these links by providing a detailed proposal of the functional relation between motor-states and interoception, based on empirical findings addressing the connection between both phenomena.

One line of evidence suggesting a reciprocal relationship between action representation and interoception is provided by functional neuroanatomy which postulates forward- and back-projections in both directions. In neuroanatomical mappings of interoceptive inference, the general argument is that the insula is the primary locus for comparator mechanisms signaling interoceptive prediction errors, whereas the cingulate and orbitofrontal cortex are considered key structures for interoceptive prediction signals. However, more recent proposals suggest that visceromotor areas, particularly the anterior insula cortex (AIC) and anterior cingulate cortex (ACC), collectively issue interoceptive predictions and encode prediction errors (Seth and Friston, 2016). This is supported by convergent anatomical connectivity and cytoarchitectonic patterns (Barrett and Simmons, 2015; Pezzulo et al., 2015). Based on knowledge obtained from neuroimaging studies, two pathways for direct input from interoception to action representation could be assumed.

First, the conjoint functioning of ACC and insular cortex has been linked with automatic processes such as salience processing and attentional direction (for review see, Medford and Critchley, 2010). In the context of research on action evaluation processes (as reviewed above), a co-activation has been consistently observed during error awareness (Klein et al., 2007). As previously mentioned, AIC activity as part of a wider salience network has been suspected to play a role in amplifying the neural signal associated with an erroneous action (Ullsperger et al., 2010; Harsay et al., 2012). That is, interoceptive awareness-related functions of AIC activity, including interoceptive prediction errors, may influence motor experience by tuning the salience of relevant sensory signals. Consequently – under predictive coding – more or less attention is paid to ascending prediction errors in the sensorimotor system so that they exert greater influence on higher-level processing (c.f., precision-weighting, Feldman and Friston, 2010; Shipp et al., 2013).

Second, earlier action selection processes provide another pathway for interoceptive signals to contribute to action selection and motor experience. The AIC has been implicated in intentional action decisions concerning the *what*- and the *when*-dimensions of an action, including the decision to inhibit an action (Brass and Haggard, 2010; for review see Droutman et al., 2015). In line with the insula playing a role for the anticipation of affective states preceding choice behavior (Knutson and Greer, 2008), direct influences between interoceptive and motor predictions may be assumed at stages of action planning and selection. Interestingly, during decision making processes, action selection fluency signals have also been suggested to prospectively contribute to a subjective sense of agency (Haggard and Chambon, 2012, for a review; Wenke et al., 2010). This contribution was found to be mediated by an exchange of signals between prefrontal action selection areas and the parietal cortex (Chambon et al., 2013). It could be hypothesized that interoceptive predictions during intention formation have direct effects on how feelings of selection fluency or dysfluency are processed and thereby influence the experience of selfhood. The strong interrelation between brain networks involved in action selection and affect anticipation could be a crucial route for interoceptive input to the sensorimotor system. Therefore, rather than indicating a hierarchical relationship as suggested in models which place motor states above interoception (Seth et al., 2012), the overall pattern of neuroscientific evidence is supportive of functional reciprocity between neuronal processes underlying interoception and intentional action at different stages of action processing and outcome monitoring. Neuroanatomical evidence is backed up by cognitive and behavioral work suggesting a functional interplay between two equivalent entities. For example, work indicating that action representations can influence interoceptive states has shown that the confirmation of action predictions in a virtual environment leads to greater immersion in its reality (Gutiérrez-Martínez et al., 2011) while electro- and psychophysiological recordings as well as behavioral indications suggest that interoceptive awareness influences motor control by being a prerequisite for conscious error awareness and the implementation of remedial actions (Ullsperger et al., 2010; Wessel et al., 2011; Sueyoshi et al., 2014; Godefroid et al., 2016). Thus, existing work touching on the interaction between interoception and action does not point to an exclusively hierarchical, unilateral relationship. Instead, evidence suggests two parallel and highly interconnected processes.

### A Proposal for the Functional Links between Motor Actions and Interoception

**FIGURE 1:**
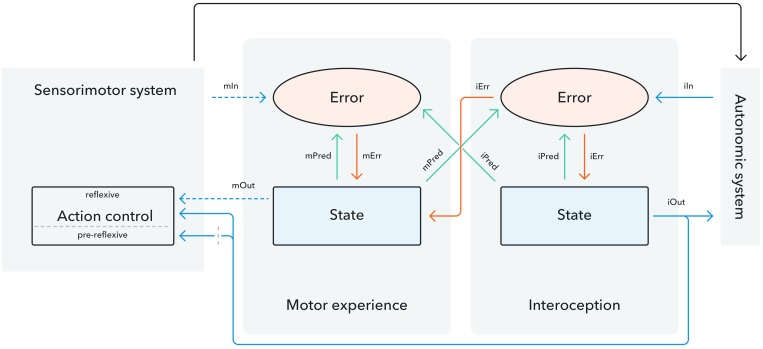
Graphical representation of the interaction between interoception and motor actions (adapted from Seth et al., 2012). Predictions relating to interoception and motor experience are generated in the state module. They are passed to the error module where they are compared to the afferent input relayed from the sensorimotor system for actions and the autonomic system for interoceptive states. The resulting error mismatch signal is sent back to the state module where it is processed to translate into a heightened or reduced sense of action ownership or interoceptive embodiment. We further propose three potential pathways in which interoceptive input could influence motor experience: (1) Interoceptive predictions [iPred] contribute to the experience of motor states. (2) The overall interoceptive state [iOut] transmits continuous information to the sensorimotor system (the overall motor state [mOut] transmits a transitory signal). (3) Continuous interoceptive information is used for reflexive forms of action control (the gray dotted barrier indicates that interoceptive input is received but is not necessary for pre-reflexive action control).

Based on the empirical and theoretical accounts discussed in this paper, we propose three specific links detailing the interaction between motor actions and interoception (see **Figure 1**), each of which forms an important avenue for future research into bodily selfhood. Our first proposal concerns the contribution of interoceptive predictions to the generation of motor states. The second link addresses the impact interoceptive signals may exert on reflexive and pre-reflexive levels of motor processing. The third link differentiates between the nature of transitory motor and continuous interoceptive signals.

First, while past accounts have theorized about the contributions of motor predictions to interoceptive states (see Seth et al., 2012 as an example) the idea of anticipated internal states affecting motor perception has remained unaddressed. We suggest a potential impact of interoceptive outcome predictions on motor experience and actions. This equates the functional significance of autonomic and sensorimotor predictions and constitutes a bi-directional link in which both entities carry equal weight in the way they contribute to and/or modulate the generation of subjective experiences such as agency and ultimately, embodied selfhood. While the above mentioned work suggests a reciprocal relationship between motor states and interoception, it should be noted that empirical findings still need to corroborate the idea of a predictive element in the generation of interoceptive states. As empirical studies with a clear focus on isolating a predictive element for interoceptive awareness are still lacking, our theory suggesting an effect of interoceptive predictions on actions is based on theoretical rather than empirical accounts. An empirical test of interoceptive inference, especially the proposed contribution of interoceptive predictions to action representations, could be achieved by inducing expectations of specific interoceptive feeling states to explore whether this affects electrophysiological markers of agency for actions in line or contrary to the expected mood. The potential verification of an impact of interoceptive predictions on motor states could lead to a more parsimonious account of the general idea of predictive coding as applied to the perception of internal and external events. Core concepts of predictive coding such as active inference (Wolpert and Flanagan, 2001; Friston et al., 2011) would now apply equally to interoceptive and motor experience. Similarly, forgone theories resting on the assumption that actions can be guided by specific feeling states they wish to achieve in the near or far future (Prinz, 2012) could likewise be integrated into the emerging theory of interoceptive inference. Evaluating the premise that actions may be guided by predicted internal states also offers the possibility of exploring the way specific motivational factors and individual differences relate to action planning, thereby leading to a more holistic, ecologically valid understanding of motor execution. Furthermore, considering interoceptive and motor states as equal entities may also contribute to a deeper understanding and, in the long term, a more effective treatment of Schizophrenia, Tourette’s Syndrome and Depersonalization Disorder in which abnormalities of action experience and interoceptive awareness often co-occur. Frequently, interoception and action experience are not impaired to the same extent and in certain cases one component is left intact. This opens the possibility of harnessing the intact dimension to alleviate symptoms associated with the malfunctioning one (i.e., by developing training to increase interoception which may improve dysfunctional motor behavior or action awareness and vice versa).

Secondly, we suggest that the contribution of the overall interoceptive state may differ for reflexive and pre-reflexive states of motor control (**Figure 1**). Based on empirical reports from the literature on error monitoring and action control, we propose that higher interoceptive awareness may lead to increased action regulation for conscious, reflexive processes performed over longer time spans while leading to more impulsive actions for pre-reflexive, transitory processes (Ainley et al., 2014). Furthermore, past theories (Seth et al., 2012) consider experiences of motor actions and interoception to be independent entities and state that input from one to the other is not necessary for either to function. Contrary to this, we argue that, while it may not be necessary for pre-reflexive forms of agency experience and motor control, interoceptive input is a prerequisite for reflexive forms of action control. We base this claim on studies showing that successful behavioral adaptation is only implemented after conscious error awareness and that conscious awareness of error commission leads to a significant increase in electrophysiological markers of error monitoring (O’Connell et al., 2007; Wessel et al., 2011; Sueyoshi et al., 2014). Empirical insight into the necessity of interoceptive input for reflexive versus pre-reflexive forms of motor control could be obtained from studying patients with lesions to brain areas implicated in specific interoceptive processes, such as the anterior cingulate cortex for interoceptive predictions (Critchley et al., 2004; Tamietto and De Gelder, 2010) or the anterior insula for the interoceptive error signal (Paulus and Stein, 2006; Palaniyappan and Liddle, 2012). Observing impairments to reflexive but not pre-reflexive forms of action control among this participant sample would corroborate theoretical accounts of agentic action control as well as offer insight into the causal relation between action representations and interoception. A line of research aiding the distinction between interoceptive input for pre-reflexive and reflexive action control could lie in exploring the autonomic correlates of (pre-reflexive) agency experiences, for which there is as yet no unequivocal evidence (cf. David et al., 2011). An interesting avenue for further research would therefore be to explicitly analyze how processes underlying autonomic regulation and action awareness operate and interact in the mind. Exploring trial-by-trial correlations between agency and markers of autonomic functioning could provide fruitful measures to distinguish between the effects of interoceptive states on conscious and pre-conscious forms of agency. Evidence for a link between interoceptive input and reflexive forms of action control may have significant implications for alleviating the symptoms of certain chronic conditions. For example, work relating heightened interoception to an increased regulatory capacity for pain highlights that treatments to increase interoception, potentially via immersion in virtual environments, could have the potential to alleviate symptoms for somatoform patients or individuals suffering from chronic pain. A possible differentiation between interoceptive input for different forms of action regulation has significant implications for our understanding of disorders in which action experience and action regulation are impaired. For example, high interoceptive accuracy is a common occurrence among highly anxious individuals (Stevens et al., 2011). These individuals have also been shown to display more impulsive and disinhibited behavior (Kashdan and Hofmann, 2008) while simultaneously demonstrating more risk aversion for long-term decisions about the self (Eisenberg et al., 1998). Considering a differential impact of interoceptive awareness on different forms of action regulation could provide insight on such behavioral patterns and lead to a deeper understanding of factors underlying action regulation.

Thirdly, the idea of predictive coding rests on the premise that an overall experience of selfhood and associated motor experiences are achieved by comparing predictions against afferent sensory feedback. We propose that the signals arising from this comparison (i.e., the error signal) may fulfill distinctly different roles in the generation of subjective motor experience depending on whether they transmit interoceptive or action-related information. Past work by Damasio (2003) argues that constant transmission of signals which reflect the body’s internal, private states is necessary for the construction of selfhood and ultimately consciousness. A sense of the self as an entity in time and space is considered an antecedent of motor experiences such as action ownership and action authorship (i.e., agency) (Blanke and Metzinger, 2009). Furthermore, Prinz (2012) provides a compelling theoretical argument in which he highlights the necessity of continuous bodily signals for conscious, agentic forms of action-control. Prinz (2012) distinguishes between two kinds of action control: a more basic, animate control of movement which is driven by former inner or outer circumstances and an evolved, agentic form of action control which, akin to the idea of active inference, is geared toward generating future anticipated outcomes. Animate action control is considered a transitory, state based process in which actions are generated in a bottom-up fashion to match the demands of an event. Conversely, agentic control is a longer-lasting, plan-based process in which actions are selected to produce a specific future goal. Prinz argues that a prerequisite for agentic action control is a continual awareness of the self as an active agent pursuing intentions and goals within a given environment. This raises an important point, as motor signals are by nature transient and thus do not possess the continuous format needed to achieve prolonged, agentic action control. The uninterrupted input needed for prolonged and continuous motor experience and action regulation can therefore only be guaranteed by the continuous stream of interoceptive signals. Prinz thus considers interoceptive input toward the formation of agency as the building block for implementing top–down, action regulation geared toward achieving desired outcomes in the near or far future and makes a convincing argument for the necessity of direct interoceptive input for long-lasting forms of conscious action control. He thereby emphasizes that the uninterrupted stream of interoceptive information is necessary for conscious forms of agentic action control as this top–down regulatory approach which is often conducted over prolonged time spans cannot be achieved by transitory motor signals.

Based on this, we suggest that the motor experience error signal is the product of transitory motor signals relayed from the sensorimotor system and thus short-lived, while the interoceptive error signal is the result of continuous visceral information about internal bodily states transmitted by the autonomic nervous system. In line with Prinz’ argument, we thus suggest that conscious agentic forms of action control require this continuous input and are therefore reliant on interoceptive input. Thus, while the sensorimotor system may not require input from the interoceptive unit for pre-reflexive forms of action control, we suggest that continuous interoceptive input is required for conscious forms of agentic action control. An empirical contribution highlighted by the continuous nature of interoceptive input for motor actions would lie in a detailed exploration of how interoception relates to action selection, action completion and agency attribution. Based on findings that fluency during action selection leads to greater agency experience (Haggard and Chambon, 2012), future work could explore whether changed autonomic responses and brain activity in the anterior cingulate and anterior insula cortex during a scenario of uncertain action selection relate to reduced feelings of agency. Similarly, the work of Allen et al. (2016) has demonstrated that unexpected bodily arousal affects confidence in perceptual decisions. If paired with measures of interoceptive awareness, their work provides a promising basis to explore whether unexplained bodily arousal reduces interoceptive awareness and whether this subsequently underpins the observed reduction of individuals’ confidence and precision regarding agency judgments. Findings to this effect would provide further evidence on the exact nature of the way in which continuous interoceptive input contributes to experiences of agency. A similar outcome would be achieved by investigating how the extent of interoceptive awareness, for example by measuring anterior insula activity, relates to feelings of action completion. Studies corroborating the necessity of continuous interoceptive input for long-lasting forms of action execution offer a potential account of the way in which we construct a seamless phenomenological experience of selfhood into which we integrate transitory motor signals. The link between interoception and action regulation indicates that a heightened or lowered awareness of internal states significantly impacts on the perception of and interaction with the external environment. Promoting and training individuals’ interoceptive capacity could thus significantly improve social interaction and safe navigation of a busy and taxing daily environment. Additionally, the possibility that interoceptive signals may be integral to long-term action planning provides a new angle to exploring individual differences concerning decision making for phenomena such as delayed gratification (Mischel et al., 1989) and reward discounting. Interestingly, discounting of delayed reward is more pronounced among individuals displaying addictive behaviors (Kirby et al., 1999; MacKillop et al., 2011) who have also been reported to show altered AIC activity thought to reflect aberrant interoceptive processing (Paulus and Stein, 2010). Findings to this effect highlight the importance of investigating whether interoceptive input may affect long-term action regulation among both normal and pathological population samples.

### The Social Construction of Conscious Interoceptive and Agentic Experience

To date, the literature discussed in this paper does not provide an indication about the processes that may contribute to the development of frameworks necessary for the conscious experience of motor actions and interoception. However, Prinz (1990, 2003, 2005, 2006) offers a comprehensive theoretical account of how the framework for conscious self-related cognition may be constructed. Considering this paper’s focus on the interaction between action and interoception, a brief look at these mechanisms is warranted as they simultaneously provide an indication of the primary link between both components. In Prinz’ (2012) view, the formation of internal architectures for motor actions and intention, as well as for self-related cognition are the product of an interaction between external social mirrors and internal mirror like representational devices. These allow an individual to arrive at a conscious understanding of self by seeing their actions and internal states reflected in others. The central premise of this idea is that individuals use their own body and mind to mirror others’ motor actions and affective states during social encounters (Tronick, 1989; Lakin et al., 2003; Brass and Heyes, 2005; Kring and Moran, 2008). This interaction is thought to contribute to the formation of internal action schemas used to match one’s own actions and affective states to those of others via a common coding mechanism for perception and action (Prinz, 1997; Hommel et al., 2001). These action schemas are subsequently used for the understanding of action volition, the development of a coherent self-structure as well as the ability to mentalize and engage in affect-regulation (Gergely et al., 2002; Fonagy and Luyten, 2009). They thus form the building blocks used to represent conscious self-generated motor actions and feeling states (Gergely et al., 2002; Southgate et al., 2009; Paulus, 2012) and constitute the underlying mechanism of the phenomenological states discussed in the above sections. The parallel evolution of action schemas used for action and interoception via the shared medium of social interactions thus forms a further indication of the strong link between both components. It further implies a moderating influence of social factors by suggesting that the social mirroring and social regulation process is a contributing factor toward generating interoceptive- and motor-related feeling states which contribute to a conscious experience of selfhood. However, empirical evidence exploring the impact of social factors on agentic and interoceptive states is still lacking and a detailed theoretical evaluation of their contribution lies outside the scope of this paper.

## Conclusion

In this paper, we provide an overview of the interaction between interoception and motor actions. Empirical and theoretical accounts of this link suggest a functional interplay in which motor and interoceptive contributions carry equal weight and have the potential to reciprocally impact on motor and interoceptive feeling states to generate a conscious experience of selfhood. Here we conceptualize this relationship by suggesting three specific links between interoception and motor actions. Firstly, we implicate interoceptive predictions in the generation of motor experience. Secondly, we distinguish between reflexive and pre-reflexive forms of motor control and the way in which interoceptive input affects each. Thirdly, we advocate the necessity of continuous interoceptive input for conscious forms of agentic action control.

A functional link between interoception and motor-actions has been considered and even implicitly assumed in the burgeoning field of research exploring the self-attribution of action and internal states in light of embodied selfhood. Research on the precise nature of this link as well as the neural network supporting it is still at its very beginnings. However, the anterior insular cortex and the anterior cingulate cortex appear to be critical underlying brain structures. Furthermore, motor actions have been found to modulate interoceptive responses, and vice versa, and recent empirical evidence seems to suggest that interoceptive input has different effects on reflexive and pre-reflexive forms of action states. The direction of these effects may vary depending on the continuous or transitory nature of efferent and afferent signals. The assumption of a bidirectional, functional connection between motor actions and interoception provides a novel theoretical angle from which to study the emergence of selfhood by exploring the functional relationship between its constituents. Empirical work to this effect has the potential to pave the way toward more ecological forms of research which captures the way our experience of motor states and our sense of internal bodily states interact with one another to interface with the environment and generate the bodily foundation of phenomenal selfhood both in health and disease. Research along these lines may thus provide new methods for characterizing subjective experiences and may also, in the long run, help to combine scientific approaches in health, social and personality psychology.

## Author Contributions

AM conducted the review and wrote the paper; AG and SS-B wrote the paper.

## Conflict of Interest Statement

The authors declare that the research was conducted in the absence of any commercial or financial relationships that could be construed as a potential conflict of interest.
